# Factors associated with health related quality of life of patients with stroke in Sri Lankan context

**DOI:** 10.1186/s12955-020-01388-y

**Published:** 2020-05-08

**Authors:** Pramudika Nirmani Kariyawasam, Kithsiri Dedduwa Pathirana, Don Chandana Hewage

**Affiliations:** 1 Department of Nursing, Faculty of Allied Health Sciences, University of Ruhuna, Galle, Sri Lanka; 2 Department of Medicine, Faculty of Medicine, University of Ruhuna, Galle, Sri Lanka; 3 Department of Physiology, Faculty of Medical Sciences, University of Sri Jayewardenepura, Gangodawila, Sri Lanka

## Abstract

**Background:**

Stroke is a major global health concern which affects the health related quality of life (HRQOL). As the prevalence of stroke is increasing especially in lower-middle income countries, it is vital to identify the factors associated with the HRQOL of affected individuals.

Available literature for post stroke HRQOL and the associated factors are mainly from high income countries. Therefore, we conducted this study to identify the factors associated with HRQOL of stroke survivors using a stroke specific measure.

**Methods:**

A longitudinal study was conducted with the participation of 257 stroke survivors. Participants were followed up after 3 months at the neurology and medical clinics in the Teaching Hospital, Karapitiya, Sri Lanka.

Health related quality of life was assessed using the validated version of Stroke Aphasia Quality of Life (SAQOL)-39 generic scale. Pearson correlation, independent sample t-test, one-way ANOVA and regression analysis were used to identify the factors associated with quality of life.

**Results:**

Mean age of the participants with stroke was 66.1 (SD 11.7) years. The mean overall HRQOL was 3.15 (SD 0.96) as measured by the SAQOL-39 g. The socio-demographic factors which had significant associations with HRQOL were; gender, level of education, marital status, occupation and monthly income (*p* < 0.05). The clinical factors which had significant associations with HRQOL were; level of dependence and disability, type of stroke, side of the lesion, type of aphasia, level of language impairment, receiving physiotherapy and speech therapy and follow up care (*p* < 0.05). The results of regression indicated six independent predictors [F (6,234) = 42.6, *p* < 0.05], with an R^2^ of 0.52. The HRQOL was significantly predicted by the level of dependence (β = .43, *p* < .01), level of language impairment (β = .20, *p* < .01), age (β = −.23, *p* < .01), type of stroke (β = −.19, *p* < .01), side of the lesion (β = .17, *p* < .01) and the level of education (β = .12, *p* < .05).

**Conclusion:**

Severe degree of dependence, severe level of language impairment, older age, hemorrhagic stroke, and lesions in the left side were associated with lower HRQOL. Higher education level was associated with higher HRQOL scores.

## Background

Stroke is the second leading cause of mortality and the third leading cause of disability worldwide [1]. It is a major global health concern that affects the quality of life of affected individuals [2, 3]. The burden of stroke is expected to increase especially in the lower-middle income countries with the current epidemiological transition of diseases [4, 5]. In Sri Lanka, stroke was the 7th major cause of in-hospital deaths in 2017 [6]. It is the leading cause of adult disability in the country and the risk of stroke is also increasing with the rising aged population [7]. Stroke related disabilities affect the individual persons, their families and finally the economy of the country. Stroke survivors encounter challenges to restore their health status within the limitations of the residual impairment [8].

Quality of life (QOL) is an important health care issue following a stroke due to concurrent disabilities [9]. Health-Related Quality of Life (HRQOL) reflects the “impact of a health state on a person’s ability to lead a fulfilling life” [10]. Stroke can affect the physical, psychological and social aspects of life while it has been associated with lowering the HRQOL [11, 12]. The factors that are associated with HRQOL of stroke survivors were identified by different authors using different assessment tools and had found different factors.

### Demographic factors associated with HRQOL

In Sri Lanka, only a few studies have been conducted to identify the factors affecting QOL of stroke survivors. According to a recent study, younger age, higher income, and better health infrastructure were associated with higher pre stroke QOL. Moreover, younger age, female gender, lower health infrastructure, lower level of education, the higher level of disability, and hypercholesterolemia were associated with lower post stroke QOL with relevant to different domains of QOL [13]. According to Ranawaka et al. age > 65 years, female gender and severity of the stroke were significant predictors of both level of disability and level of dependence in stroke survivors [14]. Although a few studies had been conducted locally, those studies were mainly focused on the QOL rather than the HRQOL. Research studies had been conducted globally to assess the HRQOL and the associated factors of the stroke survivors.

A study conducted in Hong Kong had found that gender and marital status as non-modifiable risk factors associated with HRQOL, they found that females had obtained lower HRQOL than males for the physical domain [15]. A European study demonstrated women had better life satisfaction than males while those without an occupation had lower satisfaction compared with individuals who had an occupation [16]. According to Dayapoglu and Tan, increasing age was associated with lower HRQOL of stroke survivors [17]. Further, age, level of education and income were factors associated with the HRQOL [12]. According to Serda et al., demographic properties such as gender and education were the predictors of QOL in patients with stroke [18].

### Clinical factors associated with HRQOL

According to Dayapoglu and Tan, the QOL was significantly associated with the side of the brain lesion but not with the type of stroke [17]. Stroke related impairments; especially upper limb paresis can affect the activities of daily living (ADL) [19]. The level of dependency on ADL and the level of disability at the time of discharge were associated with poor QOL in patients with stroke [14, 20]. Further, Kwok et al. found that higher Barthel Index (BI) scores had a positive correlation with the physical and psychological domains of QOL [15]. The level of disability of patients which was measured by the Modified Rankin Scale (MRS) was one of the independent determinants associated with lower HRQOL [9]. Further, stroke related impairment and functional disabilities were identified as important predictors of functional QOL of stroke survivors [21, 22]. Language impairments as aphasia had been identified as a factor that affects the QOL. According to Hilari, patients with aphasia obtained significantly lower post stroke QOL scores than those without aphasia [23]. The QOL of stroke survivors was significantly different based on the degree of aphasia [24]. Ross and Wertz compared the QOL of patients with and without aphasia and they found that social relationships, level of dependence and environment aspects were significantly different between the two groups [25]. Moreover, depression was also identified as a factor that can reduce the HRQOL of stroke survivors [8, 21, 22]. Having comorbid NCDs as hypercholesterolemia and diabetes were identified as predictors of post stroke QOL in previous studies [14, 26].

Based on previous literature, expert knowledge and experiences gained by treating stroke survivors, we aimed to assess the association of different variables that can affect the post-stroke HRQOL. Socio-demographic factors, clinical factors as type of stroke, level of disability and dependence, rehabilitation and follow-up care, comorbidities, etc. were assessed as variables of HRQOL.

The final outcome of post-stroke care and rehabilitation is to improve the HRQOL of patients. Therefore, findings of this study will be important to improve the HRQOL of the patients by identifying the associated factors. A few studies were conducted in Sri Lanka to assess the QOL and associated factors among stroke survivors. Those studies did not use disease specific measures to determine the QOL of the participants. Therefore, in our study, the Stroke Aphasia Quality of Life − 39 generic (SAQOL-39 g) scale was used to assess the post stroke HRQOL which assesses the QOL under three main domains; physical, communication and psychosocial.

### Purpose of the study

This study aimed to assess the factors associated with HRQOL of stroke survivors in Sri Lanka. The findings of this study will be vital when planning and evaluating the outcomes of rehabilitation interventions.

## Methods

### Study design and sample

A longitudinal study was conducted at the neurology and medical clinics in the Teaching Hospital, Karapitiya and it is the main tertiary care center in Southern Sri Lanka. The diagnosis of stroke was based on clinical diagnosis by treating medical officers and supported by the computerized tomographic scan. Convenient sampling method was used and all the patients with stroke including patients with or without aphasia, admitted to the neurology and medical wards of the hospital from May 2016 to May 2017 were assessed initially. Of all 368 patients, 300 patients were recruited based on inclusion/exclusion criteria. Among 300 participants, we were able to follow up 257 patients after 3 months of the onset of the stroke in the follow-up clinics. The medical records were also evaluated during the follow-up visit. All the patients were community-dwelling at the time of follow up. The patients were evaluated by a registered nurse using validated questionnaires. Ethical approval for the study was obtained from the ethics review committee, faculty of Medicine, University of Ruhuna (ref. 26.05.2015:3.15) and the written informed consent was obtained from the patient or a selected proxy (immediate family member, close friend).

Subjects with the first ever stroke, age between 18 and 80 years, who were conscious were recruited. Patients with behavioural or memory problems, patients with major psychiatric illnesses or any other neurological disorders, chronic ethanol abuse or narcotic abuse, who are on medication which can affect cognition, and subjects with mental retardation and learning disabilities were excluded from the study.

### Study instruments and timing of assessment

Socio-demographic characteristics (age, gender, marital status, monthly income, level of education, occupation), clinical characteristics (level of dependence on ADL, level of disability, level of language impairment, type of stroke, side of the lesion, having NCDs), rehabilitation (receiving physiotherapy, speech therapy and occupational therapy, Ayurvedic treatment) and details regarding follow up care received by the patients were assessed using an interviewer-administered questionnaire. The variables in the questionnaire were decided based on the previous literature, expert knowledge, and experience gained by the authors. The validated Sinhala version of the Mississippi Aphasia Screening Test (MAST) was used to detect aphasia among study participants. The level of language impairment and type of aphasia (expressive, receptive or global) were also assessed using the MAST based on the total score and subscale scores. The Sinhala version of the MAST is a psychometrically sound screening test to detect aphasia which consists of two main indices and nine subscales. The score range is 1–100 and higher scores indicated better language ability [27].

The validated Sinhala version of the SAQOL-39 g scale was used to assess the HRQOL of the participants. The scale has been translated and validated into the Sinhala language and showed excellent internal consistency and validity [28]. The HRQOL of patients with severe aphasia (those who could not self-report) was evaluated with the proxy-rated version of the Sinhala SAQOL-39 g. The SAQOL-39 g scale can be used to assess the HRQOL of the stroke survivors with or without aphasia [29]. The validated Sinhala version contains 39 items under three main domains as physical, psychosocial and communication. Each item has a 5 point scoring sheet and at the end of the assessment, the overall mean score and the domain mean scores can be calculated. The maximum mean score that a patient can obtain is 5 (range 1–5). The Sri Lankan validation of the Barthel Index (BI) was used to determine the level of dependence [30]. The level of dependence on daily living was assessed using the validated Sri Lankan version of the Barthel Index (BI). It has been identified as a reliable and valid test to evaluate the activities of daily living. There are items to assess the ability for feeding, grooming, bathing, dressing, bowel and bladder care, toilet use, ambulation, transfers, and stair climbing. The maximal score 100 indicates that the patient is fully independent in physical functioning. The Modified Rankin Scale (MRS) was used to determine the level of disability. It has been used in research studies and recommended to assess the level of disability among stroke survivors [31].

The socio-demographic characteristics, clinical characteristics and the level of dependence and disability were assessed during hospitalization before discharge. Details regarding follow-up care and rehabilitation interventions were obtained after 3 months of the onset of the stroke at the follow-up clinic.

### Data analysis

The Statistical Package of Social Sciences (SPSS) version 20 was used to analyze data. Kolmogorov-Smirnov test was used to assess the normality of dependent variables (HRQOL overall score, subdomain scores). The results related to 257 patients who were able to follow up were analysed. Pearson correlation (continuous variables as age, BI score, and MAST score), and independent sample t-test (gender, type of stroke, side of the lesion, received rehabilitation) were used to determine the association of those factors with the HRQOL. One-way ANOVA and post hoc test (Bonferroni) (categorical variables as the level of education, marital status, income, occupation, type of aphasia) were conducted to find the associations with the HRQOL of stroke survivors. The factors which were significantly associated were further analysed using multiple linear regression. The weak correlations were excluded by applying the stepwise manner to identify the independent predictors of HRQOL of patients with stroke. The *p-*value of < 0.05 was considered significant.

## Results

### Socio-demographic characteristics of the patients

The mean age of the participants with stroke (*n* = 257) was 66.1 (SD 11.7) years (range 33–80) of which 58.8% (*n* = 151) were males. Most of the patients, 48.6% (*n* = 125) had no monthly income in the pre-stroke period and were depending on their family members. Of all patients, 16.8% (*n* = 43) were educated up to advanced level and 39.7% (*n* = 102) had only primary education. The majority of the patients, 75.9% (*n* = 195) were married.

### Clinical characteristics of the patients

The proportion of the ischemic stroke was 80.9% (*n* = 208) while 19.1% (*n* = 49) had hemorrhagic strokes. According to the Kolmogorov-Smirnov test, the HRQOL were distributed normally (*p* > 0.05). The overall mean HRQOL was 3.15 (SD 0.96) as measured by the SAQOL-39 g. Moreover, mean scores for physical, psychosocial and communication domains were 3.17 (SD 0.96), 3.11 (SD 0.97) and 3.35 (SD 0.98) respectively. The mean score for the MAST was 71.4 (SD 34.2) out of 100. There were 31.9% (*n* = 82) patients who had aphasia, of which, 16% (*n* = 41) had global aphasia while 13.6% (*n* = 35) and 2.3% (*n* = 6) had expressive and receptive aphasia respectively.

The BI mean score after 3 months at the follow up was 64.7 (SD 26.4), which was significantly higher (*p* < 0.05) compared to BI score at the acute stage in the hospital. The most prevalent comorbid NCD was hypertension (65%) followed by diabetes mellitus (31.1%) and dyslipidemia (36.2%).

### Socio-demographic factors associated with post stroke HRQOL

Age was negatively correlated with overall HRQOL (*r* = −.31), *p* < 0.01 as shown in Table 1.

**Table 1:** Correlations between HRQOL Mean Scores and Age, Barthel Index Score, MAST Score,(*n* = 257)

Factor	Overall	Physical	Communication	Psychosocial
Age (*r) p*-value	.-31	−.26	−.19	−.33
	0.001^**^	0.002^*^	0.002^**^	.0.001^**^
Barthel index score (*r*) *p* value	.58	.56	.36	.48
	0.001^**^	0.001^**^	0.001^**^	0.001^**^
MAST score (*r*) *p* value	.47	.39	.60	.41
	0.001^**^	0.001^**^	0.001^**^	0.001^**^

** Pearson correlation is significant at the 0.01 level (2-tailed)

The socio-demographic factors which had significant associations with HRQOL were; gender, level of education, marital status, occupation and monthly income (*p* < 0.05) (Table 2). Males obtained a significantly higher overall score (*M* = 3.25, SD 1) than females (*M* = 2.98, SD 0.9) t (238) = (2.1), *p* < 0.05.

**Table 2:** Socio-demographic Factors associated with Post Stroke HRQOL of Life, *n* = 257

Factor	Physical	Communication	Psychosocial	Overall
**Gender**	Mean (*SD*)			
Male (*n* = 151)	3.27 (1)	3.34 (1.2)	3.21 (1)	3.23 (1)
Female (*n* = 106)	3.01 (0.7)	3.37 (0.7)	2.95 (0.9)	2.98 (0.9)
*t*	2.0	−0.1	2.1	2.1
*p*-value	0.046*	0.895	0.039*	0.035*
**Level of Education**	Mean (*SD*)			
Primary Education (*n* = 102)	3.1 (0.9)	3.0 (1)	2.8 (0.8)	2.9 (0.8)
Up to Ordinary Level (*n* = 112)	3.2 (1)	3.5 (1)	3.2 (0.9)	3.2 (0.9)
Up to Advanced Level (*n* = 43)	3.4 (1.1)	3.7 (1)	3.4 (1)	3.5 (1)
*F*	1.9	6.4	6.7	5.3
*p*-value	0.157	0.002**	0.01**	0.005**
**Marital status**				
Married (*n* = 195)	3.3 (1)	3.4 (1)	3.2 (0.9)	3.2 (0.9)
Unmarried (*n* = 26)	3.2 (1)	3.2 (1)	3.0 (1)	3.1 (1)
Widowed/Separated (*n* = 36)	2.7 (1)	3.1 (1)	3.0 (1)	2.7 (0.8)
*F*	3.6	0.9	0.8	3.4
*p*-value	0.03*	0.40	0.47	0.03*
**Monthly Income**	Mean (*SD*)			
< Rs.10,000 (*n* = 206)	3.1 (1)	3.2 (1)	3.0 (0.9)	3.0 (0.8)
Rs.10,000–30,000 (*n* = 31)	3.3 (1)	3.3 (1)	3.3 (1)	3.3 (0.9)
> Rs.30,000 (*n* = 20)	3.2 (1)	4 (1)	3.9 (1)	3.9 (1)
*F*				
*p*-value	0.001**	0.042*	0.001*	0.001**
**Occupation**	Mean (*SD*)			
No occupation (*n* = 77)	2.8 (0.9)	2.2 (0.9)	3.1 (1)	2.9 (0.8)
Business (*n* = 41)	3.7 (0.9)	3.7 (0.9)	3.7 (0.8)	3.7 (0.9)
with occupation (*n* = 85)	3.0 (1)	3.3 (1)	3.0 (1)	3.0 (1)
Retired (*n* = 54)	3.5 (0.7)	3.6 (1)	3.2 (0.8)	3.4 (0.7)
*F*	9.2	2.5	7.6	8.4
*p*-value	0.001**	0.06	0.001**	0.001**

* Statistically significant at the level*-p* < 0.01

* Statistically significant at the level*-p* < 0.05

One way ANOVA was conducted to determine the effect of the level of education, marital status, occupation and monthly income on HRQOL of the patients with stroke. The HRQOL was significantly different based on the level of education, *F* (2, 254) = 5.4, *p* < 0.01 and those with a higher level of education had better HRQOL. The HRQOL was also significantly associated with the marital status of the patients, *F* (2, 254) = 3.4, *p* < 0.05. Those who married obtained higher mean scores for the overall as well as subdomains of the SAQOL-39 g scale.

The overall HRQOL was significantly associated with the monthly income of the participants in the pre-stroke period, *F* (2,254) = 9.1, *p* < 0.01. Higher monthly income was associated with better HRQOL. The HRQOL was statistically different based on the occupation *F* (2, 254) =8.4, (*p* < 0.01). Those who were engaged in their own business and retired had better HRQOL compared to others.

### Clinical characteristics associated with post stroke HRQOL

The HRQOL was significantly correlated with the level of dependence (*r* = 0.58, *p* < 0.01) and with the level of language impairment of patients which was assessed using the MAST (*r* = 0.47, *p* < 0.01). The correlations between variables are shown in Table 1.

The associations of clinical characteristics with HRQOL are shown in Table 3.

**Table 3:** Clinical Factors Associated with the Post Stroke HRQOL, *n* = 257

Factor	Physical	Communication	Psychosocial	Overall
**Level of disability**	Mean (*SD*)			
Slight disability (*n* = 53)	3.8 (0.8)	3.7 (1)	3.6 (0.9)	3.7 (0.8)
Moderate disability (*n* = 58)	3.6 (0.9)	3.7 (1)	3.5 (0.8)	3.6 (0.8)
Moderately severe disability (*n* = 93)	2.9 (0.8)	3.1 (1)	2.8 (0.7)	2.9 (0.7)
Severe disability (*n* = 93)	2.5 (1)	2.9 (1)	2.6 (1)	2.5 (1)
*F*	28.2	7.1	18.9	26.4
*p* value	0.001^**^	0.001^**^	0.001**	0.001^**^
**Type of stroke**				
Ischemic (*n* = 208)	3.31 (1)	3.62 (1)	3.24 (0.9)	3.32 (0.9)
Haemorrhagic (*n* = 49)	2.52 (0.9)	2.53 (1)	2.62 (0.9)	2.52 (0.8)
*t*	5.0	5.6	4.6	5.3
*p*-value	0.001**	0.005**	0.001**	0.001**
**Side of the lesion**				
Left hemisphere (*n* = 161)	2.92 (1)	2.81 (1)	2.9 (0.9)	2.92 (1)
Right hemisphere (*n* = 95)	3.32 (0.9)	3.62 (1)	3.31 (0.3)	3.32 (0.9)
*t*	3.6	5	3.1	3.6
*p*-value	0.001**	0.003**	0.002**	0.001**
**Type of Aphasia**				
No aphasia (*n* = 154)	3.4 (0.9)	3.8 (1)	3.3 (0.9)	3.4 (0.9)
Global aphasia (*n* = 41)	2.5 (0.9)	2.1 (0.8)	2.5 (0.6)	2.4 (0.7)
Expressive aphasia (*n* = 35)	2.9 (0.9)	2.4 (0.9)	2.8 (0.8)	2.7 (0.8)
Receptive aphasia (*n* = 6)	3.0 (0.9)	3.4 (0.9)	3.0 (1.0)	3.0 (0.9)
*F*	11.4	42.1	11.9	17.7
*p*-value	0.001**	0.001**	0.001**	0.001**
**Follow-up care**				
Regular (*n* = 171)	3.22 (0.9)	3.46 (1)	3.23 (0.9)	3.28 (0.9)
Not regular (*n* = 86)	3.0 (1)	3.26 (1)	3.0 (1)	3.0 (1)
*t*	2.2	1.4	1.8	2
*p*-value	0.03*	0.16	0.07	0.05*

* Statistically significant at the level*-p* < 0.01

* Statistically significant at the level*-p* < 0.05

Patients with no/slight disability obtained higher scores for all the domains of HRQOL than those who had moderate/ severe disability levels *F* (3,253) = 26.4, *p* < 0.01.

Patients with ischemic strokes had significantly higher overall scores (*M* = 3.3, SD 0.9) than patients with hemorrhagic strokes (*M* = 2.5, SD 0.8) at *t* (255) = 5.3, *p* < 0.05.

The HRQOL was also significantly associated with the type of aphasia, *F* (3,253) = 17.7, *p* < 0.01. Patients without aphasia had better HRQOL than patients with aphasia. Those with global aphasia had lower HRQOL (*M* = 2.4, SD 0.8) than patients with expressive aphasia (*M* = 2.7, SD 0.8) and receptive aphasia (*M* = 3, SD 0.9). The HRQOL was significantly associated with the side of the lesion. Patients with left hemisphere lesions (*M* = 2.9, SD 1) had obtained lower scores than patients with right hemisphere lesions (*M* = 3.3, SD 0.8) and it was statistically significant, *t* (254) = 3.6, *p* < 0.05.

Having NCDs such as hypertension, diabetes mellitus and dyslipidemia were analysed. Not having any NCD, having only one NCD and having two or more NCDs were not significantly associated with the HRQOL (*p* > 0.05).

Patients who received speech therapy obtained significantly lower scores (*M* = 2.8, SD 0.9) than those who did not receive speech therapy (*M* = 3.2, SD 0.9), *p* < 0.05. Those who had referred and participated for physiotherapy obtained significantly lower HRQOL scores (*M* = 2.9, SD 0.8) than those who did not receive (*M* = 3.3, SD 1), *p* < 0.05. Those who participated for regular follow up obtained higher scores for HRQOL than those who did not attend follow up (*p* < 0.05).

### Significant predictors of the post stroke HRQOL

The associated variables were further analysed using Regression analysis. The results of multiple linear regression showed six independent predictors [F (6,234) = 42.6, p < 0.05], with an R^2^ of 0.52 for the overall HRQOL score. The overall HRQOL was significantly predicted by the level of dependence, level of language impairment, age, and type of stroke, side of the lesion and the level of education. The domain scores were also analysed to identify the significant predictors for each domain (The results of regression analysis are shown in Table 4).

**Table 4:** Independent Predictors of Post Stroke HRQOL, (*n* = 257)

	Significant predictors	Standardized beta coefficient	*p* value	Adjusted R^2^
Overall HRQOL	Level of dependence (BI score)^c^	.43^b^	0.001	0.52
	Level of language impairment (MAST score)	.20^b^	0.001	
	Age	−.23^b^	0.001	
	Type of stroke	−.19^b^	0.001	
	Side of the lesion	.17^b^	0.002	
	Level of education	.12^a^	0.022	
Physical domain	Level of dependence (BI score)^c^	.43^b^	0.001	0.44
	Level of language impairment (MAST score)	.18^b^	0.001	
	Age	−.23^b^	0.001	
	Type of stroke	−.17^b^	0.001	
	Side of the lesion	.15^b^	0.001	
Communication domain	Level of language impairment	.46^b^	0.001	0.52
	Level of dependence	.46^b^	0.001	
	Type of stroke	−.16^b^	0.001	
	Side of the lesion	.19^b^	0.001	
	Level of education	.12^b^	0.009	
	Level of disability	.31^b^	0.002	
	age	−.18^b^	0.001	
	Occupation	.17^b^	0.001	
Psychosocial domain	Level of dependence	.33^b^	0.001	0.40
	Level of language impairment	.22^b^	0.001	
	Age	.29^b^	0.001	
	Type of stroke	−.17^b^	0.001	
	Side of the lesion	. 13^b^	0.012	

^a^Significant at the 0.05 level (2-tailed)

^b^Significant at the 0.01 level (2-tailed)

^c^The ability to perform Activities of Daily Living based on Barthel Index scores

## Discussion

This study was conducted to determine the factors associated with HRQOL of stroke survivors. We identified socio-demographic as well as clinical care factors that are associated with the HRQOL of study participants. The higher level of dependence, severe language impairments, older age, haemorrhagic stroke, and left side lesions were associated with lower HRQOL. The higher level of education was associated with higher HRQOL.

The overall HRQOL, physical, communication, and psychosocial domains were predicted by the age of stroke survivors. A previous Sri Lankan study had found that older age was significantly associated with lower post stroke QOL [14]. Similarly, advancing age demonstrated lower HRQOL in stroke survivors [18, 22]. This may be due to the age-related functional decline and aging itself can create functional disabilities that affect the HRQOL. As the population aging is in an upward trend, the policymakers should pay special attention when establishing stroke units. Those units should be designed particularly for the needs of the elderly people and health care team members should be trained to fulfill the required needs of aged stroke survivors.

Although gender and marital status were not significant predictors in regression analysis, those factors showed a significant association with the overall HRQOL. Interpretations on HRQOL were confirmed with post-hoc analysis. The gender was significantly associated with physical and psychosocial domains, while marital status was associated with the overall HRQOL and physical domain. Females had lower HRQOL scores than males, which is in line with previous studies [15, 22]. The mean age of the females (69.1 years) was significantly higher than the age of the male stroke survivors (64 years). This may be one of the reasons for lower QOL as increased age affects the QOL. In contrast, Baumann et al. demonstrated women had better life satisfaction than men after 2 years of onset of stroke [16]. Marital status had significant association with the HRQOL which was also in line with a previous study [21]. Those who were married had obtained higher scores than unmarried and widowed/separated patients. The reason may be the support from the family members or spouse improves the recovery from the disabilities of stroke than those who do not receive support from the family. Therefore, patients who need further social support should be evaluated at the time of discharge and during follow-up visits.

The overall HRQOL, physical, communication, and psychosocial domains were significantly different based on the level of education and income. Previous studies also found similar findings regarding the level of education and income [13, 18]. The level of education of the participants can affect the compliance of the treatment and management guidelines as they may have a lack of understanding of the recovery process. Furthermore, a better education means higher income and occupation. On the other hand, poor income may be a barrier for seeking proper follow up when needed. Patients with good income may have more transport facilities, better access to health care services than others. Therefore, health education programmes for patients to improve awareness regarding treatment and follow-up care would improve the HRQOL.

In our study, the level of dependence which was measured using the BI was the most significant predictor of overall HRQOL and the other three domains. The QOL was lower when the patient is more dependent on others for ADL as shown with previous studies. The QOL was significantly associated with the level of dependence of patients with stroke [13, 15]. Similarly, Kwok et al., also showed that increasing BI score had a positive correlation with physical and psychological HRQOL [15]. When the patient can carry out his/her day to day activities alone or with minimal assistance, their perception of QOL will be better. Establishing hospital and community based post stroke rehabilitation centres in the country to improve the daily living activities will be helpful for a better HRQOL.

The type of stroke and side of the lesion were significant predictors of overall HRQOL as well as all three domains. Those who had hemorrhagic strokes had lower mean scores than patients with ischemic strokes. The reason may be that in our study those with hemorrhagic stroke had lower Barthel index scores compared to patients with ischemic stroke. Dayapoglu and Tan had found the QOL was significantly different based on the side of the lesion but not with the type of stroke [17]. Those who had right hemisphere lesions obtained better scores than patients with left hemisphere lesions. Functional impairment of the dominant limbs limits the ADL of patients with left hemisphere lesions than patients with right hemisphere lesions. Moreover, patients with left hemisphere lesions had language difficulties as aphasia which can be associated with lower HRQOL.

Although comorbid NCDs do not significantly associate with the HRQOL in our study, having NCDs as hypercholesterolemia and diabetes were identified as predictors of post stroke QOL in previous studies [13, 26]. According to a previous study, there was a significant positive association with mobility improvement after physiotherapy treatments [32]. In contrast, we found that HRQOL was negatively associated with receiving physiotherapy and speech therapy. That is when compared with those who received physiotherapy and speech therapy had lower HRQOL scores than those who did not. This was not as expected, because receiving physiotherapy and speech therapy should improve the HRQOL scores of the patients. In our clinical settings, most of the patients who referred and participated in physiotherapy and speech therapy had more disabilities compared to others. Although patients mentioned they participated in physiotherapy and speech therapy interventions, it may be not regular. Moreover, we did not assess the frequency and intensity of rehabilitation interventions. Future studies should be focused on the effect of rehabilitation interventions on the HRQOL. Moreover, there is a need to investigate novel rehabilitation interventions for stroke survivors in Sri Lanka using new technology.

There were several limitations in the present study such as using a proxy rated version of the SAQOL-39 g scale to assess the HRQOL of patients with severe language impairments. A nominated proxy was interviewed on behalf of the patient in an effort of including all the stroke survivors without excluding those with severe language disorders. There can be a difference between the proxy’s perception and the patient’s perception as some domains such as personality and mood are highly subjective. Further, we evaluated the HRQOL of the participants 3 months after the stroke. Out of 300 patients, only 257 had participated in follow-up clinics. The two groups (participants and missing group to follow up) were not significantly different from age, gender, level of education, income.

The level of cognition and the presence of psychological disorders such as depression and anxiety may have a significant effect on HRQOL of stroke survivors but, we did not assess them in the current study due to lack of standardized test batteries specifically developed and validated for stroke survivors in Sri Lankan context. Appropriate test batteries should be developed by the researchers to determine the HRQOL of stroke survivors with communicative and cognitive impairments. Moreover, studies should be conducted to determine the level of cognition, the prevalence of psychological disorders such as depression among patients with aphasia using validated tests in Sri Lanka. These factors may also have an association with the HRQOL of stroke survivors. Further, qualitative studies can be conducted to assess the psychological and social factors and how those factors affect the HRQOL of the patients. As the neurorehabilitation is an emerging specialty in Sri Lanka, the policymakers, health care team members should consider the factors that we have identified in our study.

## Conclusion

The level of dependence, level of language impairment, age, type of stroke, side of the lesion and level of education were independent predictors of post stroke HRQOL. Higher levels of dependence, increasing age, hemorrhagic strokes and left-sided lesions were associated with lower HRQOL, while a higher level of education was associated with better HRQOL in this study population.

### Recommendations

The factors associated with the HRQOL of patients with stroke need to be taken into account when planning rehabilitation interventions. Future studies should be conducted in the Sri Lankan context to determine the level of cognition, the prevalence of psychological disorders such as depression using validated tests in patients with stroke, as these factors may also have an association with the HRQOL of patients with stroke.

## Data Availability

The data sets used and/or analyzed during the current study are available from the corresponding author on reasonable request.

## Abbreviations

ADL: Activities of Daily Living
BI: Barthel Index
HRQOL: Health Related Quality of Life
MAST: Mississippi Aphasia Screening Test
MRS: Modified Rankin Scale
NCD: Non Communicable Diseases
QOL: Quality of Life
SAQOL-39 g scale: Stroke Aphasia Quality of Life-39 generic scale

## References

[1] Lozano R, Naghavi M, Foreman K, Lim S, Shibuya K, Aboyans V, Abraham J, Adair T, Aggarwal R, Ahn SY, AlMazroa MA (2012). Global and regional mortality from 235 causes of death for 20 age groups in 1990 and 2010: a systematic analysis for the global burden of disease study 2010. Lancet.

[2] Feigin VL, Krishnamurthi RV, Parmar P, Norrving B, Mensah GA, Bennett DA, Barker-Collo S, Moran AE, Sacco RL, Truelsen T, Davis S (2015). Update on the global burden of ischemic and hemorrhagic stroke in 1990-2013: the GBD 2013 study. Neuroepidemiology..

[3] Mayo NE, Wood-Dauphinee S, Coˆte R, Durcan L, Carlton J (2002). Activity, participation, and quality of life 6 months poststroke. Arch Phys Med Rehabil.

[4] Feigin VL, Lawes CM, Bennett DA, Barker-Collo SL, Parag V (2009). Worldwide stroke incidence and early case fatality reported in 56 population-based studies: a systematic review. Lancet Neurol.

[5] Mukherjee D, Patil CG (2011). Epidemiology and the global burden of stroke. World Neurosurgery.

[6] Annual Health Bulletin, Indoor Morbidity Mortality Report (2014). Ministry of Health Sri Lanka.

[7] Wijeratne T, Gunaratne P, Gamage R, Pathirana G, Senanayake S, De Silva N, Sirisena D, Wijegunasinghe D. Stroke care development in Sri Lanka: the urgent need for Neurorehabilitation services. Neurology Asia. 2011;16(2).

[8] Bays CL (2001). Quality of life of stroke survivors: a research synthesis. J Neurosurg Nurs.

[9] Abubakar SA, Isezuo SA (2012). Health related quality of life of stroke survivors: experience of a stroke unit. Int J Biomed Sci.

[10] Carr AJ, Gibson B, Robinson PG (2001). Is quality of life determined by expectations or experience?. Bmj..

[11] Haley WE, Roth DL, Hovater M, Clay OJ (2015). Long-term impact of stroke on family caregiver well-being: a population-based case-control study. Neurology..

[12] Jeon NE, Kwon KM, Kim YH, Lee JS (2017). The factors associated with health-related quality of life in stroke survivors age 40 and older. Ann Rehabil Med.

[13] Mahesh PK, Gunathunga MW, Jayasinghe S, Arnold SM, Liyanage SN (2018). Factors influencing pre-stroke and post-stroke quality of life among stroke survivors in a lower middle-income country. Neurol Sci.

[14] Ranawaka U, Alexander F, Nawaratne D, Liyanage H, Kulatunga A, Tissera N, Cooray M, Kasthuriratne A, Wickramasinghe R. Factors Affecting Early Outcome in Sri Lankan Patients with Stroke (P06. 234). http://n.neurology.org/content/78/1_Supplement/P06.234.short.

[15] Kwok T, Lo RS, Wong E, Wai-Kwong T, Mok V, Kai-Sing W (2006). Quality of life of stroke survivors: a 1-year follow-up study. Arch Phys Med Rehabil.

[16] Baumann M, Couffignal S, Le Bihan E, Chau N. Life satisfaction two-years after stroke onset: the effects of gender, sex occupational status, memory function and quality of life among stroke patients (Newsqol) and their family caregivers (Whoqol-bref) in Luxembourg. BMC Neurol 2012; 12(1):105. doi: 10.1186%2F1471-2377-12-105. 10.1186/1471-2377-12-105 PMC3551740 23009364

[17] Dayapoglu N, Tan M (2010). Quality of life in stroke patients. Neurol India.

[18] Serda EM, Bozkurt M, Karakoc M, Çağlayan M, Akdeniz D, Oktayoğlu P, Varol S, Kemal NA (2015). Determining quality of life and associated factors in patients with stroke. Turk Soc Phys Med Rehabil.

[19] Veerbeek JM, Kwakkel G, van Wegen EE, Ket JC, Heymans MW (2011). Early prediction of outcome of activities of daily living after stroke: a systematic review. Stroke..

[20] Sturm JW, Donnan GA, Dewey HM, Macdonell RA, Gilligan AK, Srikanth V, Thrift AG (2004). Quality of life after stroke: the north East Melbourne stroke incidence study (NEMESIS). Stroke..

[21] Jaracz K, Jaracz J, Kozubski W, Rybakowski JK (2002). Post-stroke quality of life and depression. Acta Neuropsychiatrica.

[22] Carod-Artal J, Egido JA, González JL (2000). Varela de Seijas E. quality of life among stroke survivors evaluated 1 year after stroke: experience of a stroke unit. Stroke..

[23] Hilari K (2011). The impact of stroke: are people with aphasia different to those without?. Disabil Rehabil.

[24] Hilari K, Wiggins R, Roy P, Byng S, Smith S (2003). Predictors of health-related quality of life (HRQL) in people with chronic aphasia. Aphasiology..

[25] Ross K, Wertz R (2003). Quality of life with and without aphasia. Aphasiology..

[26] Oni OD, Ojini FI, Olisah VO, Aina OF (2016). Quality of life and associated factors among poststroke clinic attendees at a university teaching Hospital in Nigeria. Niger Med J.

[27] Kariyawasam PN, Pathirana KD, Hewage DC (2016). Validation of the Sinhala version of Mississippi Aphasia Screening Test (MAST), Proceedings of 5th Biennial Conference of South Asian Association of Physiologists.

[28] Kariyawasam PN, Pathirana KD, Hewage DC, Dissanayake RDA (2017). (2017), Validation of Sinhala version of stroke aphasia quality of life −39 generic (SAQOL-39g) scale, proceedings of academic sessions (IPURSE).

[29] Hilari K, Lamping DL, Smith SC, Northcott S, Lamb A, Marshall J. Psychometric properties of the stroke and aphasia quality of life scale (SAQOL-39) in a generic stroke population. Clin Rehabil 2009; 23(6):544 doi: 10.1177%2F0269215508101729. 10.1177/0269215508101729 19447841

[30] Lekamwasam S, Karunatilake K, Lekamwasam V. Physical dependency of elderly and physically disabled; measurement concordance between 10-item Barthel index and 5-item shorter version. Ceylon Med J. 2011;56(3). 10.4038/cmj.v56i3.3603 22164749

[31] Broderick JP, Adeoye O, Elm J (2017). Evolution of the modified Rankin scale and its use in future stroke trials. Stroke.

[32] Paolucci S (2008). Epidemiology and treatment of post-stroke depression. Neuropsychiatr Dis Treat.

